# Mesenchymal Mycn participates in odontoblastic lineage commitment by regulating *Krüppel-like Factor 4* (*Klf4*) in mice

**DOI:** 10.1186/s13287-022-02749-8

**Published:** 2022-02-22

**Authors:** Zhuo Huang, Ruihuan Yang, Ruyi Li, Yining Zuo, Fan Gu, Miao He, Zhuan Bian

**Affiliations:** The State Key Laboratory Breeding Base of Basic Science of Stomatology (Hubei-MOST) and Key Laboratory of Oral Biomedicine Ministry of Education, School and Hospital of Stomatology, Wuhan University, Wuhan, Hubei China

**Keywords:** Odontoblast lineage, Transcription factors, Dental biology, μCT, Genetic animal models

## Abstract

**Background:**

Commitment of mouse dental papilla cells (mDPCs) to the odontoblast lineage is critical for dentin formation, and this biological process is regulated by a complex transcription factor network. The transcription factor Mycn is a proto-oncogene that plays an important role in tumorigenesis and normal embryonic development. An early study revealed that *Mycn* is exclusively expressed in dental mesenchymal cells at E15.5, which implies a potential role of *Mycn* in dentinogenesis. However, the role of *Mycn* in dentin formation remains elusive. Thus, it is of considerable interest to elucidate the role of *Mycn* in dentin formation.

**Methods:**

*Mycn*^*fl/fl*^; *Osr2*^*IresCre*^ (*Mycn*^*Osr2*^) and *Mycn*^*fl/fl*^; *K14*^*Cre*^ (*Mycn*^*K14*^) transgenic mice were generated, and micro-CT scans were performed to quantitatively analyse the volumetric differences in the molars and incisors of the mutants and their littermates. *Mycn* was also knocked down in vitro*,* and alkaline phosphatase (ALP) and alizarin red staining (ARS) were conducted. Cleavage under targets and tagmentation (CUT&Tag) analysis and dual luciferase assays were performed to identify direct downstream targets of Mycn. Immunofluorescence and immunochemistry staining and western blotting (WB) were performed to analyse the expression levels of potential targets. Quantitative PCR, WB, ALP and ARS were performed to test the rescue efficiency.

**Results:**

Mesenchymal ablation of *Mycn* (*Mycn*^*Osr2*^) led to defective dentin formation, while epithelial deletion (*Mycn*^*K14*^) had no obvious effects on tooth development. ALP and ARS staining revealed that the commitment capacity of mDPCs to the odontoblast lineage was compromised in *Mycn*^*Osr2*^ mice. CUT&Tag analysis identified *Klf4* as a potential direct target of Mycn, and a dual luciferase reporter assay verified that Mycn could bind to the promotor region of *Klf4* and directly activate its transcription. Reciprocally, forced expression of *Klf4* partially recovered the odontoblastic differentiation capacity of mDPCs with *Mycn* knockdown.

**Conclusions:**

Our results elucidated that mesenchymal Mycn modulates the odontoblastic commitment of dental papilla cells by directly regulating *Klf4*. Our study illustrated the role of Mycn in dentin development and furthers our general comprehension of the transcription factor networks involved in the dentinogenesis process. Thus, these results may provide new insight into dentin hypoplasia and bioengineered dentin regeneration.

**Supplementary Information:**

The online version contains supplementary material available at 10.1186/s13287-022-02749-8.

## Introduction

Tooth development depends on the succession of reciprocal interactions between the oral ectoderm and neural crest-derived ectomesenchyme [1]. The superficial ectoderm produces the hardest tissue enamel, whereas the underlying ectomesenchyme builds up another mineralized tissue dentin. Dentinogenesis is executed by the differentiation and mineralization of odontoblasts. Studies have shown that dental pulp cells, even in adult teeth, can still differentiate into odontoblast-like cells under appropriate stimulation [2, 3]. However, cells derived from other nondental mesenchymal stem cells do not possess such potential, indicating that the differentiation of dental mesenchymal stem cells into odontoblast lineages is cell type-specific. Therefore, understanding the molecular mechanisms underlying the commitment of dental mesenchymal stem cells to the odontoblast lineage is of great significance.

Numerous studies have shown that the commitment of dental papilla cells, odontoblast precursors, to the odontoblast lineage is governed by several signalling pathways, such as Wnt/β-catenin [4], bone morphogenetic protein (BMP) [5], sonic hedgehog (SHH) [6] and fibroblast growth factor (FGF) [7, 8]. In recent years, the role of transcription factors, including Krüppel-like Factor 4 (Klf4) [9, 10], Osterix (Osx or Sp7) [11–13], GATA-binding protein 4 (Gata4) [14, 15] and Runt-related transcription Factor 2 (Runx2) [16, 17], in odontoblast commitment has also been emphasized. Transcription factors function in cell fate determination and cell differentiation by binding to the promoter regions of target genes to activate or repress their expression. Transcription factor networks can feasibly orchestrate signalling cascades manipulating odontoblast lineage commitment. However, the mechanisms underlying this process are still elusive.

The transcription factor Mycn is a member of the Myc proto-oncogene family (comprising Myc, Mycn, Mycl), which plays crucial roles in modulating cell proliferation, differentiation, apoptosis and survival during tumorigenesis and normal embryonic development [18, 19]. Targeted elimination of *Mycn* resulted in embryonic lethality at mid-gestation [20]. Tissue-specific conditional knockouts also demonstrated that *Mycn* is essential for specific developing organs where Mycn is highly expressed during embryonic development. In mouse lung progenitor cells, for example, Mycn expression was observed in the distal population of undifferentiated epithelial cells, and conditional deletion in these cells led to severely malformed lungs [21]. In addition, in our previous study, *Mycn* deletion in cranial neural crest cells (*Mycn*^*Wnt1−Cre*^) generated abnormal offspring resembling the Pierre Robin sequence (PRS) in humans with cleft palate, microglossia and micrognathia [22], indicating that Mycn is also crucial for craniofacial development. In addition, studies assessing *Mycn* levels in developing mouse tooth buds showed that *Mycn* transcripts were exclusively detected in dental papilla cells at E15.5 [23], which implied that *Mycn* may also participate in the dentinogenesis process. However, to the best of our knowledge, studies on the potential roles of *Mycn* in dental development are limited. Thus, it is of considerable interest to elucidate the role of *Mycn* in dental formation. Here, we investigated the role of *Mycn* in dentinogenesis by inducing the mesenchymal inactivation of *Mycn* under the control of the *Osr2* promoter and investigated its role in enamel formation by inducing the epithelial ablation of *Mycn* under the control of the *K14* promoter. Our results demonstrated that while epithelial deletion of *Mycn* in dental germ cells had no effects on enamel development, mesenchymal *Mycn* could control the odontoblastic differentiation of dental papilla cells and thus impact dentin development in mice by directly regulating *Klf4* transcription.

## Materials and methods

### Animals

The animals used in the study included *Mycn*^*fl/*+^ transgenic mice (JAX#006933; The Jackson Laboratory, Bar Harbour, ME, USA), *Osr2*^*IresCre*^ mice (JAX#009388; The Jackson Laboratory), *K14*^*Cre*^ mice (Shanghai Model Organisms Center, Shanghai, China), *ROSA*^*mT/mG*^ mice (JAX#007676; The Jackson Laboratory) and CD1 wild-type mice. The genotypes of the transgenic mice were determined as previously described [24]. To specifically knock *Mycn* out in dental mesenchymal cells, *Mycn*^*fl/fl*^ mice were crossed with *Mycn*^*fl/*+^;*Osr2*^*IresCre*^ mice to generate *Mycn*^*Osr2*^ transgenic mice. To specifically knock *Mycn* out in dental epithelial cells, *Mycn*^*fl/fl*^ mice were crossed with *Mycn*^*fl/*+^;*K14*^*Cre*^ mice to generate *Mycn*^*K14*^ transgenic mice.

### Osr2^+^ and K14^+^ cell lineage tracing

To analyse the Cre activity of *Osr2*^*IresCre*^ and *K14*^*Cre*^ mice, the mice were crossed with *ROSA*^*mT/mG*^ mice. The embryonic heads were dehydrated in 30%, 50% and 90% sucrose solutions for 1 h at room temperature, embedded in optical cutting temperature (OCT) compound (Tissue-Tek; Sakura Finetek USA, Inc., Torrance, CA, USA) and cryosectioned at 10 μm using a cryostat (CM1950; Leica Biosystems Inc., USA).

### Histology, immunohistochemistry and immunofluorescence

*Mycn*^*Osr2*^ (*Mycn*^*fl/fl*^; *Osr2*^*IresCre*^) and control (*Mycn*^*fl/fl*^ or *Mycn*^*fl/*+^; *Osr2*^*IresCre*^) mice were harvested at E13.5-E18.5 and P0. Control and mutant embryonic heads were fixed in 4% paraformaldehyde (PFA) at 4 °C overnight, and mandibles were then decalcified in 10% ethylenediaminetetraacetic acid (EDTA) for 1–2 weeks. Samples were dehydrated in serial concentrations of ethanol, embedded in paraffin and sectioned at 5 μm using a microtome (RM2255; Leica Biosystems Inc., USA). For histologic analysis, deparaffinized sections were stained with haematoxylin and eosin (H&E) using standard procedures. Antigen retrieval was achieved by boiling sections in Citrate-EDTA Antigen Retrieval Solution (Beyotime Biotechnology, Shanghai, China) for 20 min in a microwave. To eliminate nonspecific binding, slides were incubated in endogenous peroxidase blocking buffer (Beyotime Biotechnology) for 20 min and blocked with QuickBlock™ (Beyotime Biotechnology) for 15 min at room temperature. Primary antibodies were incubated at 4 °C overnight. Slides were washed three times in phosphate-buffered saline (PBS) before incubation with secondary antibodies for 1 h at 37 °C. For immunohistochemical examination, immune complexes were visualized using a diaminobenzidine (DAB) kit (Maixin, Fuzhou, China).

### Micro-CT and X-radiography (X-ray) analysis

Mandibles were dissected at PN6W, fixed in 4% PFA and scanned with a micro-CT system (μCT-50; SCANCO Medical AG, Brüttisellen, Switzerland) with an isotropic resolution of 7 μm at 55 kVp and 200 μA. Generated ISQ files were imported into ImageJ to acquire image sequences. Next, the exported images were analysed using Mimics software (Materialise, Leuven, Belgium). The enamel and dentin of different groups were segmented and reconstructed separately using the same threshold values. The volumes of teeth, enamel and dentin, as well as the pulp chamber height of mandibular first molars, were calculated and compared. For X-ray analysis, mandibles were harvested at PN6M and scanned with an X-ray microanalysis system (In vivo DXS Pro; Bruker, Germany).

### 5-Bromo-2′-deoxyuridine (BrdU) incorporation assay

For cell proliferation assays, pregnant mice were injected intraperitoneally at E15.5 with BrdU (50 µg/g body weight) 4 h prior to sacrifice. Embryonic heads were then processed as described above.

### Cell cultures and odontoblastic differentiation

Mouse dental papilla cells (mDPCs) were isolated from the first molars of E15.5 CD1 mice and digested for 1 h at 37 °C in a solution of 3 mg/mL collagenase type I (Worthington Biochemical, USA) and 4 mg/mL Dispase II (Roche, Mannheim, Germany). The cells were cultured in Dulbecco’s modified Eagle’s medium (DMEM; 11995; Gibco, Grand Island, NY, USA) containing 10% foetal bovine serum (FBS; 10099141; Gibco) plus 1% penicillin and streptomycin (control medium, CM). To induce odontoblastic differentiation, mDPCs were seeded at a density of approximately 100,000 cells per well in 12-well plates and treated with odontoblastic medium (OM; CM supplemented with 10^−^7 M dexamethasone (Sigma–Aldrich, St Louis, MO, USA), 50 µg/mL ascorbic acid (Sigma–Aldrich) and 10 mM sodium β-glycerophosphate (Sigma–Aldrich)). Primary mDPCs at passages 2–4 were used for cell experiments. HEK293E cells were cultured in DMEM (Gibco) supplemented with 10% FBS. MC3T3-E1 cells were cultured in minimum essential medium (MEM Alpha basic; 12571; Gibco) supplemented with 10% FBS. Cells were cultured at 37 °C in a humidified atmosphere of air containing 5% CO_2_.

### Lentiviral transfection

A recombinant lentivirus encoding an shRNA targeting *Mycn* (*shMycn*), a lentivirus overexpressing *Mycn* and a mock lentivirus were designed and constructed by GeneChem (Shanghai, China). To overexpress Klf4, the complementary DNA (cDNA) encoding *Klf4* with the EcoRI/BamHI enzyme sites was amplified and cloned into the EcoRI/BamHI sites of the pCDH-CMV-MCS-EF1-copGFP vector (System Biosciences, CA, USA) using the ClonExpress One Step Cloning Kit (Vazyme, Nanjing, China). Then, the empty vector or pKlf4 vector was cotransfected with packaging plasmids psPAX2 and pMD2. G into HEK293E cells using Lipofectamine 3000 (Invitrogen). After 48 h, virus particles were harvested. Lentiviruses were diluted in cell culture medium with 5 μg/ml polybrene (Beyotime Biotechnology) and placed on mDPCs seeded 24 h prior to infection. To induce odontoblastic differentiation, the growth medium was replaced with OM after 4 days. Each medium was replaced every other day.

### Real-time quantitative PCR (RT–qPCR)

After the indicated incubation, mDPCs were rinsed with PBS twice, and total RNA was extracted using the RNAprep pure Cell/Bacteria Kit (TIANGEN, Beijing, China) and reverse transcribed by the RevertAid First Strand cDNA Synthesis Kit (Thermo Fisher Scientific, USA). RT–qPCR was performed with a CFX Connect Real-Time System (Bio–Rad, CA, USA) using ChamQ Universal SYBR qPCR Master Mix (Vazyme). PCR cycling conditions were 95 °C for 30 s followed by 40 cycles at 95 °C for 10 s and 60 °C for 30 s. DNA melting curves (95 °C for 10 s, 65 °C for 5 s and then 95 °C for 5 s) were routinely performed. Relative gene expression levels were normalized to those of *β*-actin using the 2^−ΔΔCT^ method.

### Western blotting

Cultured mDPCs were rinsed with PBS twice and lysed in radioimmunoprecipitation assay (RIPA) buffer followed by centrifugation at 16,000 g at 4 °C for 10 min. The concentration of total protein in the supernatant was measured using the BCA Protein Assay Kit (Pierce Biotechnology, IL, USA). Equal amounts of protein were loaded and separated on an 8% polyacrylamide gel and transferred onto polyvinylidene difluoride (PVDF) membranes (Roche). The membranes were then blocked in QuickBlock™ buffer (Beyotime Biotechnology) at room temperature for 15 min, followed by incubation with the primary antibodies. Then, membranes were incubated with secondary antibodies at room temperature for 1 h, visualized by SuperSignal West Pico PLUS (Thermo Fisher) reagents and imaged by an Odyssey CLx Imaging System (LI-COR, NE, USA).

### Cleavage under targets and tagmentation (CUT&Tag) analysis

The CUT&Tag library was prepared using the Hyperactive In-Situ ChIP Library Prep Kit for Illumina (TD901; Vazyme) according to the manufacturer’s instructions with minor revisions. Briefly, primary mDPCs at E15.5 were cultured with CM for three days as described above. Approximately 100,000 cells were used per sample, and three biological replicates were performed (Mycn_01-03). Cells were bound to concanavalin A-coated magnetic beads (TD901; Vazyme) and incubated in an anti-Mycn antibody for 2 h. After removing the primary antibody on a magnetic stand, a goat anti-rabbit IgG (Additional file 1: Table S1) was incubated for 1 h, followed by incubation with hyperactive pG-Tn5 transposon (TD901; Vazyme) for 1 h. Next, the cells were incubated with Tagmentation Buffer (TD901; Vazyme) for 1 h. The above incubations were all performed with rotation at room temperature. The DNA from the cells was extracted and purified. Libraries were dual-indexed, amplified and purified with VAHTS DNA Clean Beads (N411; Vazyme). Sequencing was performed with an Illumina NovaSeq 6000 platform (provided by Novogene Company (China), with a sequencing depth of 6G bases for each sample.

### Dual luciferase reporter assay

The *Klf4* promoter region (1 kb) containing the Mycn binding site with the MluI/HindIII enzyme sites was synthesized and inserted into the MluI/HindIII sites of the pGL6-TA luciferase vector (Beyotime Biotechnology) to construct a *Klf4*-luciferase reporter plasmid (pGL6-*Klf4*). MC3T3-E1 cells were seeded at a density of approximately 50,000 cells per well in 24-well plates. When the cells reached 80% confluence, they were cotransfected with the pGL6-*Klf4* reporter vector, a pRL-TK vector that encodes Renilla luciferase and a mock vector or the *Mycn* expression vector using Lipofectamine 3000 Transfection Reagent (Invitrogen). Firefly and Renilla luciferase activities were determined 48 h later using a dual-luciferase reporter assay system (Beyotime Biotechnology) with a luminometer (GloMax; Promega, WI, USA). Firefly luciferase activities were normalized against Renilla luciferase activities.

### Alkaline phosphatase (ALP) and alizarin red staining (ARS)

After transfection with mock lentivirus, *shMycn* lentivirus and/or pCDH-CMV-Klf4 lentivirus, mDPCs were cultured in CM for 4 days and OM for 9 or 11 days. Cells were fixed with 4% PFA and stained with a BCIP/NBT Alkaline Phosphatase Colour Development Kit (Beyotime Biotechnology) or alizarin red assay (Sigma–Aldrich). For ALP activity quantification, cells were lysed with 0.1% Triton/PBS solution, and the ALP activity was analysed with an alkaline phosphatase assay kit (Beyotime Biotechnology). Protein concentrations were determined as mentioned above. ALP activity was defined as micromoles of reaction product (*p*-nitrophenol) per minute from a mg of cellular protein [25]. For ARS quantification, 10% cetylpyridinium chloride (Aladding, Shanghai, China) solution was added to the stained cells and incubated at room temperature overnight. The solutions were then collected and measured at OD 562 nm by a microplate reader.

### Antibodies and primers

The antibodies and primers used in the study are listed in Additional file 1: Tables S1-2.

### Statistical analyses

All experiments were repeated at least 3 times. Statistical analyses were conducted using SPSS (Version 19.0; IBM, US). Since all data were normally distributed (Shapiro–Wilk test), Student’s test (two‐tailed) was applied to compare the mutants and the controls. The results are presented as the means ± standard deviation, and *p* < 0.05 was considered statistically significant.

## Results

### Mycn expression during tooth development

**Fig. 1:**
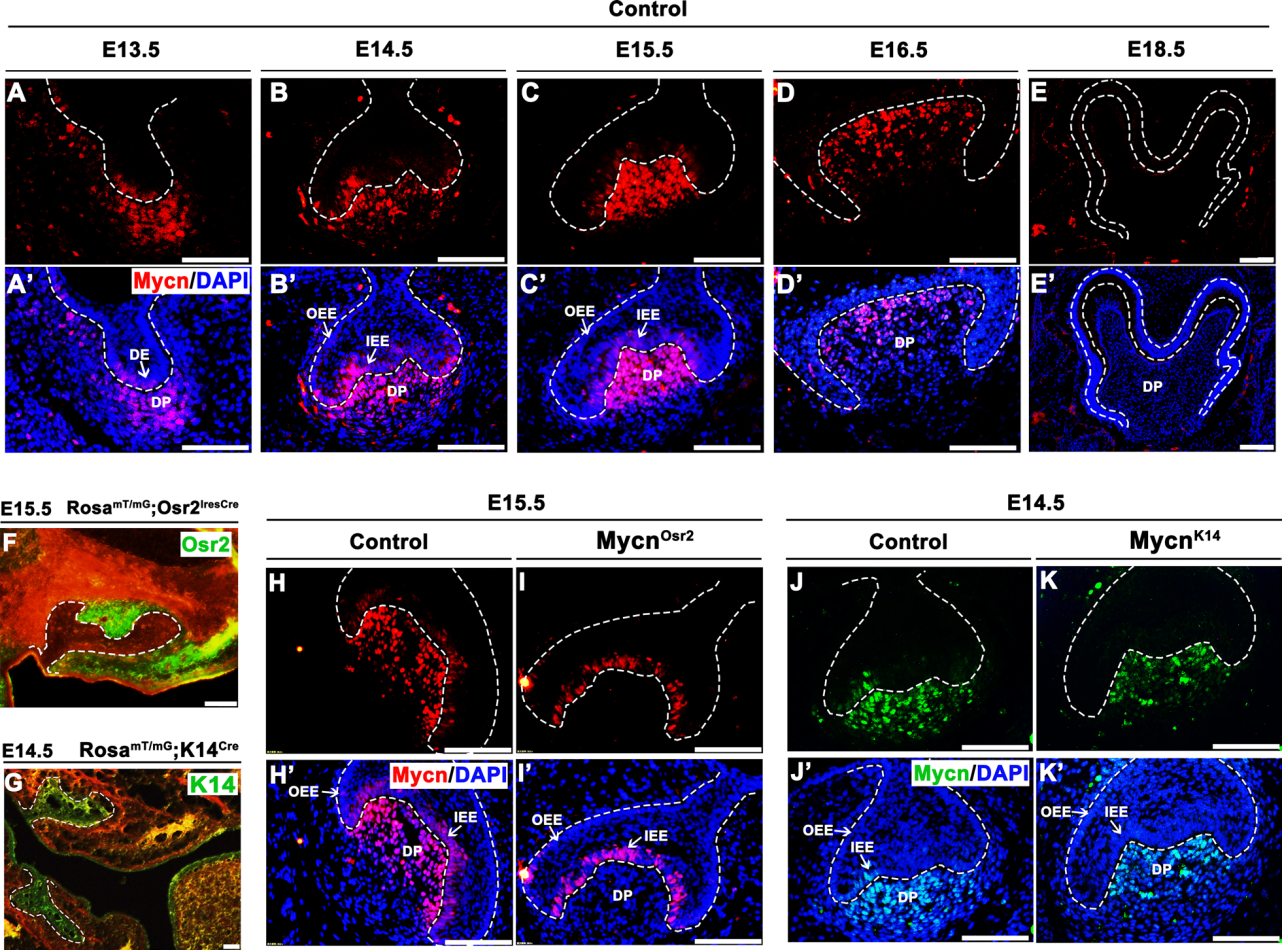
Temporospatial expression pattern of Mycn in tooth germ cells and verification of *Mycn* deletion in mutant mice. (**A–E’**) Immunofluorescence of Mycn (red) overlaid with DAPI (blue) at embryonic days 13.5 (E13.5) (**A–A’**), E14.5 (**B–B’**), E15.5 (**C–C’**), E16.5 (**D–D’**) and E18.5 (**E–E’**) of control mice. (**F–G**) Validation of the Cre activity (green) of *Osr2*^*IresCre*^ mice (**F**) at E15.5 and *K14*^*Cre*^ mice at E14.5 (**G**). (**H–I’**) Immunofluorescence of Mycn (red) overlaid with DAPI (blue) at E15.5 of control (**H–H’**) and *Mycn*^*Osr2*^ mutant mice (**I–I’**). (**J–K’**) Immunofluorescence of Mycn (green) overlaid with DAPI (blue) at E14.5 of control (**J–J’**) and *Mycn*^*K14*^ mutant mice (**K–K’**). Dotted lines indicate the epithelial compartment of the tooth germ. DE, dental epithelium; DP, dental papilla; OEE, outer enamel epithelium; IEE, inner enamel epithelium. Scale bars: 50 μm

To verify the exact temporospatial expression pattern of Mycn in tooth germ, we mapped Mycn expression from E13.5 to E18.5 by immunofluorescence. The results showed that Mycn was expressed in the tooth germ of mandibular first molars shortly after the initiation of tooth development (Fig. 1A, A’). Robust expression of Mycn was detected from E14.5 to E15.5 in dental mesenchymal cells (Fig. 1B, C’). By E16.5, mesenchymal Mycn showed slightly decreased expression levels (Fig. 1D, D’). In addition, modest expression of Mycn was also observed in dental epithelium from the bud stage to the early bell stage (Fig. 1A–C’). At the late bell stage (E18.5), Mycn expression could hardly be detected in the whole tooth germ (Fig. 1E, E’). Given that the bud-to-bell transition stage is crucial for the proliferation and differentiation of dental epithelial and mesenchymal progenitors, the extensive expression of Mycn in tooth organs during this stage suggests that Mycn may potentially be involved in the early development of enamel and dentin.

### Mesenchymal ablation of *Mycn* results in thinner dentin

Considering the dual expression of Mycn in both dental mesenchymal and epithelial cells, we generated *Mycn*^*Osr2*^ and *Mycn*^*K14*^ mice to reveal the potential effects of *Mycn* on tooth development, as the *Osr2*^*IresCre*^ and *K14*^*Cre*^ mouse strains were widely used in conditional knockout of genes expressed in oral mesenchyme [26] and epithelium [27], respectively. By crossing with the ROSA^mT/mG^ mice, the activity of the *Cre* recombinase of these two transgenic mice was validated (Fig. 1F, G). Then, we confirmed that *Mycn* was efficiently diminished in the dental mesenchymal cells of *Mycn*^*Osr2*^ mice (Fig. 1H, I’) and in the dental epithelium of *Mycn*^*K14*^ mice (Fig. 1J, K’). Unlike *Mycn* null embryos that died by E12.5, *Mycn*^*Osr2*^ and *Mycn*^*K14*^ embryos were viable and generated fertile adults.

**Fig. 2:**
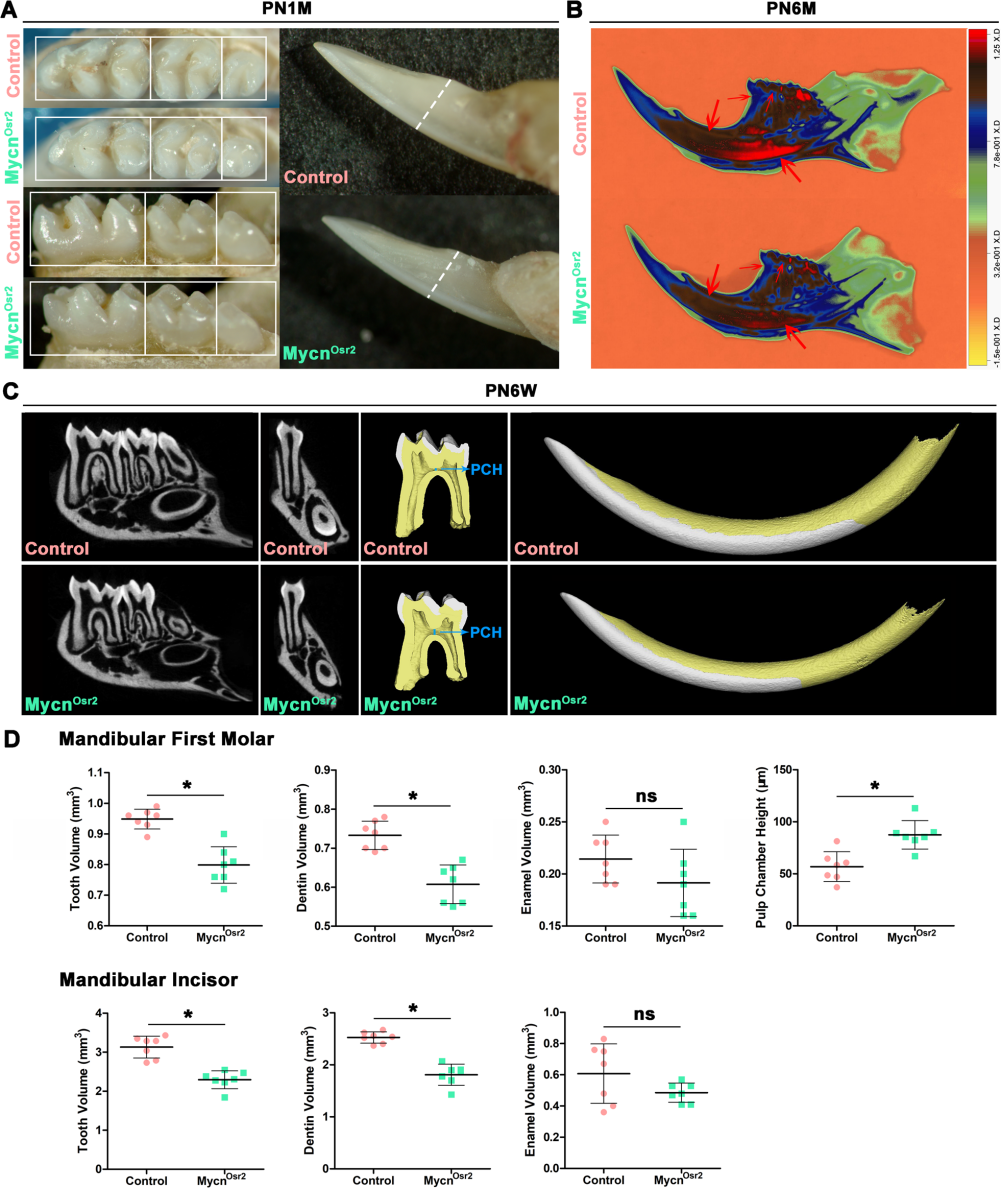
Mesenchymal ablation of *Mycn* results in thinner dentin in mandibular first molars and incisors. (**A**) Gross appearances show smaller mandibular molars and incisors of *Mycn*^*Osr2*^ mutants compared with controls at PN1M. (**B**) Heatmap analysis of the X-ray radiograph shows distinctly lower dentin density of the *Mycn*^*Osr2*^ mutant at PN6M. Red arrows point to the representative areas (the lingual side and apical area of incisor and the cementoenamel junction and dental cusp of molar) of decreased dentin greyscale. (**C**) Representative micro-CT scanning images and three-dimensional reconstructions (white: enamel; yellow: dentin) of control and mutant mice at PN6W. PCH: pulp chamber height. (**D**) Quantification of tooth, dentin and enamel volumes of mandibular first molars and incisors and pulp chamber height of the molars. *p < 0.05. *ns* not statistically significant

To reveal the phenotypic differences, we first compared the gross appearance of the mandibular molars of *Mycn*^*Osr2*^ and *Mycn*^*K14*^ mice with their littermate controls under a stereomicroscope. While the teeth of *Mycn*^*K14*^ mice appeared as normal as those of the control mice (Additional file 1: Fig. S1A-C’), the teeth of *Mycn*^*Osr2*^ mice looked distinctly smaller than those of the littermate controls at PN1M (Fig. 2A). Then, we quantitatively analysed the tooth size of all groups by micro-CT. Representative micro-CT scans and the reconstructed 3-dimensional mandibular first molars and incisors are shown in Fig. 2C. The results showed that no significant difference in tooth volumes was observed between *Mycn*^*K14*^ mice and the control littermates (Additional file 1: Fig. S1D) at PN6W. However, the volumes of mandibular first molars and incisors were significantly decreased (19% in molars; 25.8% in incisors) in *Mycn*^*Osr2*^ mutants compared with control mice at PN6W, and these dimensional losses were probably caused by a significant reduction (25% in molars; 28% in incisors) in dentin, since no significant differences were observed in enamel volumes (Fig. 2D). In addition, the pulp chamber height of mandibular first molars of the mutants was significantly higher (53%) than that of the controls, which also implied that thinner dentin was formed in *Mycn*^*Osr2*^ mutants. For dentin density analysis, high-resolution X-radiographs characterized by a heatmap were acquired at PN6M. As shown in Fig. 2B, the dentin density of both molars and incisors in *Mycn*^*Osr2*^ mice was distinctly decreased compared with that of the control. A massively decreased greyscale of dentin can readily be observed at the lingual side and apical area of the incisor and the cementoenamel junction and dental cusp of molars in *Mycn*^*Osr2*^ mice (red arrows). Taken together, our results suggest that *Mycn* is more likely to be obligate for dentinogenesis rather than enamel synthesis and that mesenchymal loss of *Mycn* is accountable for thinner and lower-density dentin and larger pulp chambers.

### Loss of *Mycn* impaired lineage commitment of dental papilla cells to odontoblasts

**Fig. 3:**
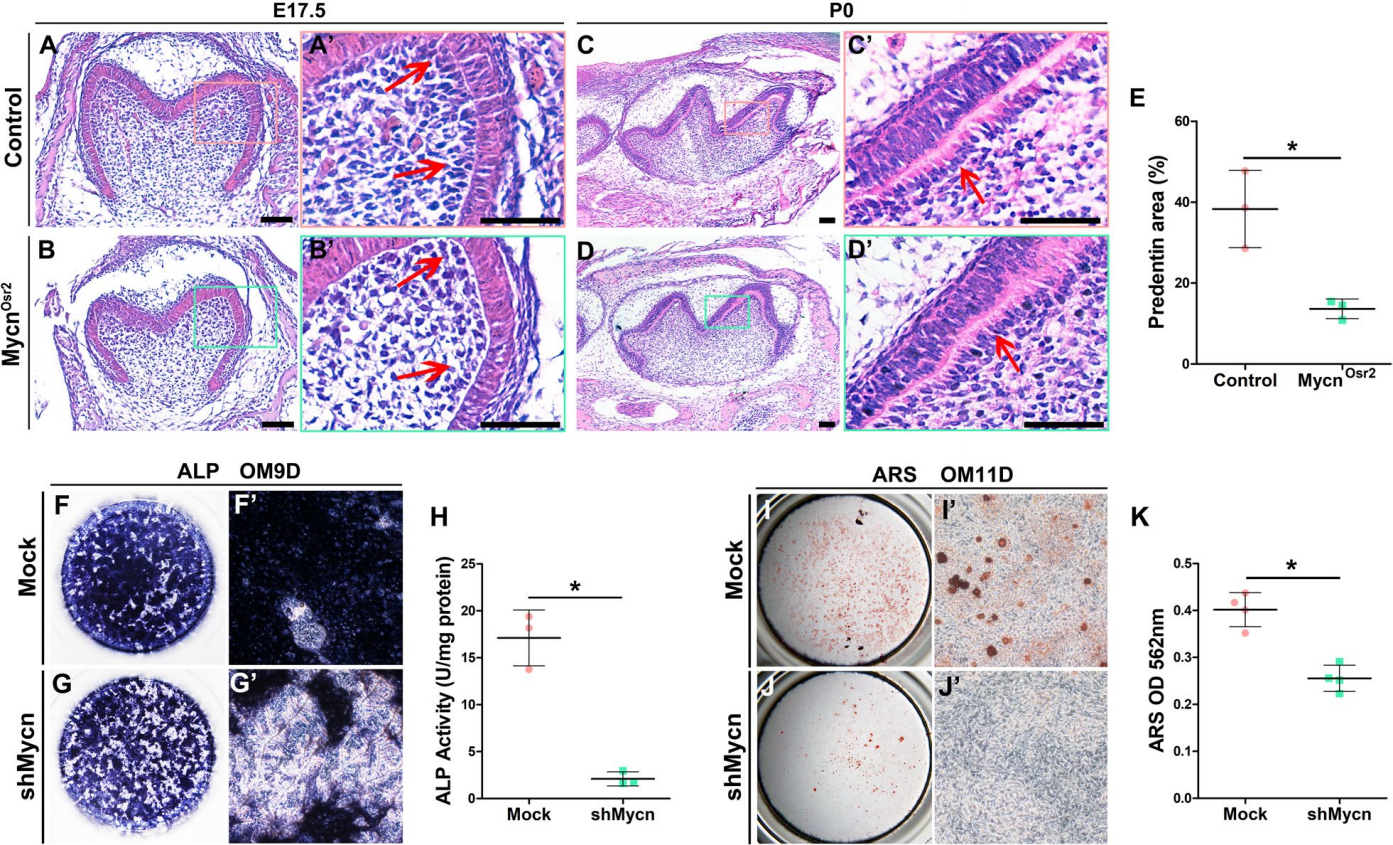
Compromised differentiation of mDPCs of *Mycn*^*Osr2*^ mutant mice. (**A–B’**) H&E staining shows fewer preodontoblasts (red arrows) in mutant mice at E17.5. (**C–D’**) H&E staining shows malformed odontoblasts (red arrows) and less predentin in mutant mice at P0. (**E**) The predentin area was significantly decreased in *Mycn*^*Osr2*^ mutant mice at P0. (**F–H**) Alkaline phosphatase (ALP) staining and quantification at 9D of odontoblastic induction show decreased ALP activity in *shMycn* mDPCs. (**I–K**) Alizarin red staining (ARS) and quantification at 11D of odontoblastic induction show decreased calcium deposition in *shMycn* mDPCs. *p < 0.05

To disclose the reason why *Mycn*^*Osr2*^ mice formed thinner dentin, we first evaluated the proliferative capacity of dental mesenchymal cells. The results of BrdU and Ki67 labelling assays both showed no significant differences between the mutants and littermate controls (Additional file 1: Fig. S2A-B’’). Immunofluorescence staining of Caspase 3 also showed no impact of *Mycn* loss on the apoptosis of dental mesenchymal cells (Additional file 1: Fig. S2C-C’’). Thereafter, we assessed the odontoblastic differentiation ability of dental papilla cells. H&E-stained slides showed that compared to the littermate controls, fewer preodontoblasts were observed in *Mycn*^*Osr2*^ mice at E17.5 (Fig. 3A, B’). In addition, *Mycn*^*Osr2*^ mice formed abnormal odontoblasts with shorter heights and less predentin by the newborn stage (Fig. 3C–E). To further verify the effect of Mycn on odontoblast lineage commitment, we knocked *Mycn* down in primary mDPCs in vitro. The results of ALP (Fig. 3F–H) and ARS staining (Fig. 3I–K) showed a significant decrease in *shMycn* cells, indicating that knockdown of *Mycn* inhibited odontoblastic differentiation and calcium deposition. This histological and staining evidence suggests that mesenchymal ablation of Mycn might obstacle the commitment of dental papilla cells to the odontoblast lineage and thus impair odontoblastic mineralization and dentin formation.

### *Klf4* is a direct downstream target of Mycn during dentinogenesis

**Fig. 4:**
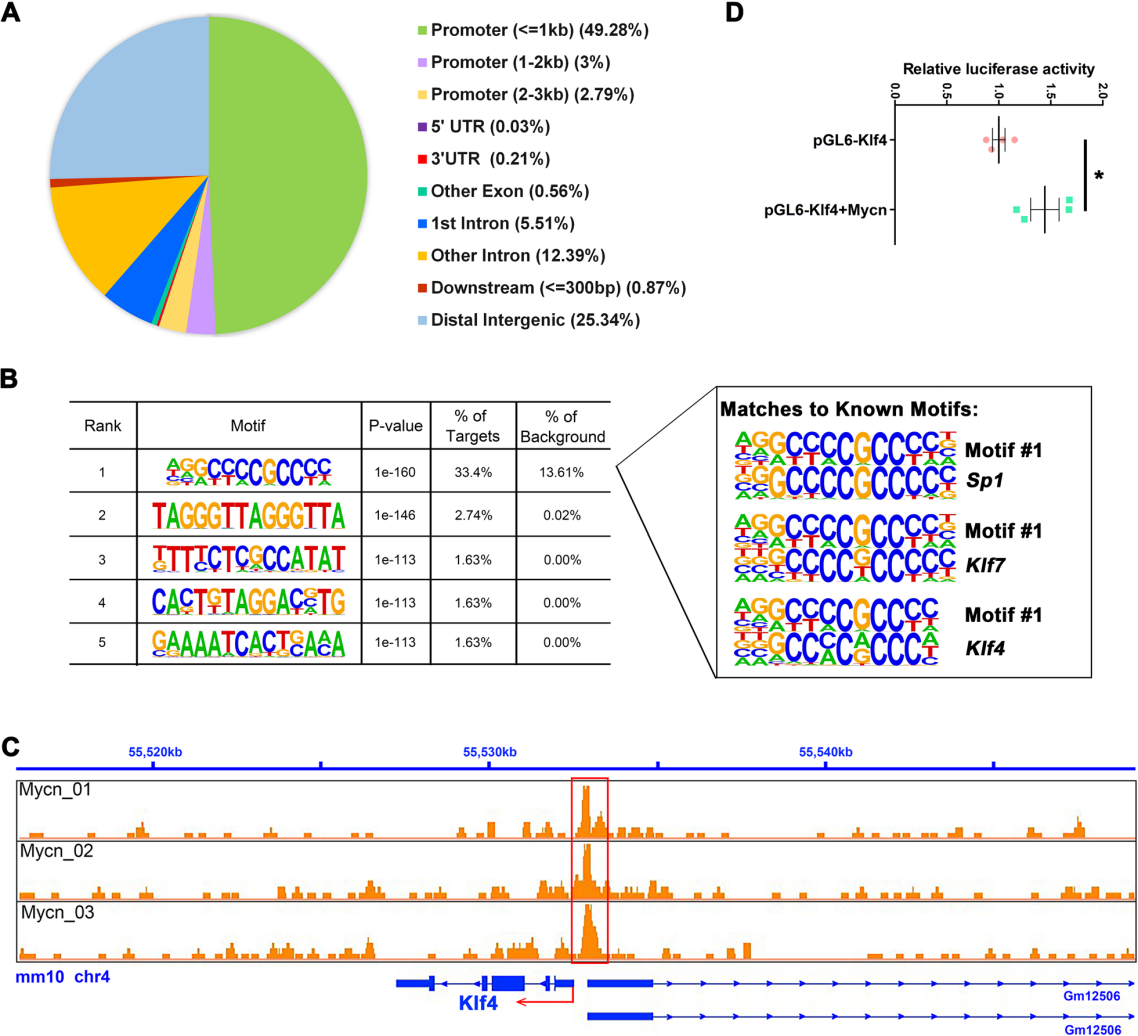
Mycn directly binds to the promoter region of *Klf4* and positively regulates its transcriptional activity. (**A**) Location of Mycn-occupancy peaks relative to the nearest annotated gene identified by CUT&Tag analysis. (**B**) Five most enriched sequence motifs at Mycn-occupancy sites as determined using HOMER. The top three matches of Motif #1 to known motifs are *Sp1*, *Klf7* and *Klf4*. (**C**) Integrative genomics viewer (IGV) screenshots show tracks of normalized Mycn-occupancy tag counts. The red box identifies peaks bound by Mycn. The red arrow denotes the transcription start site (TSS) and transcription direction. (**D**) Dual luciferase assay shows that Mycn overexpression significantly upregulated the luciferase activity of the *Klf4* promoter. *p < 0.05

**Fig. 5:**
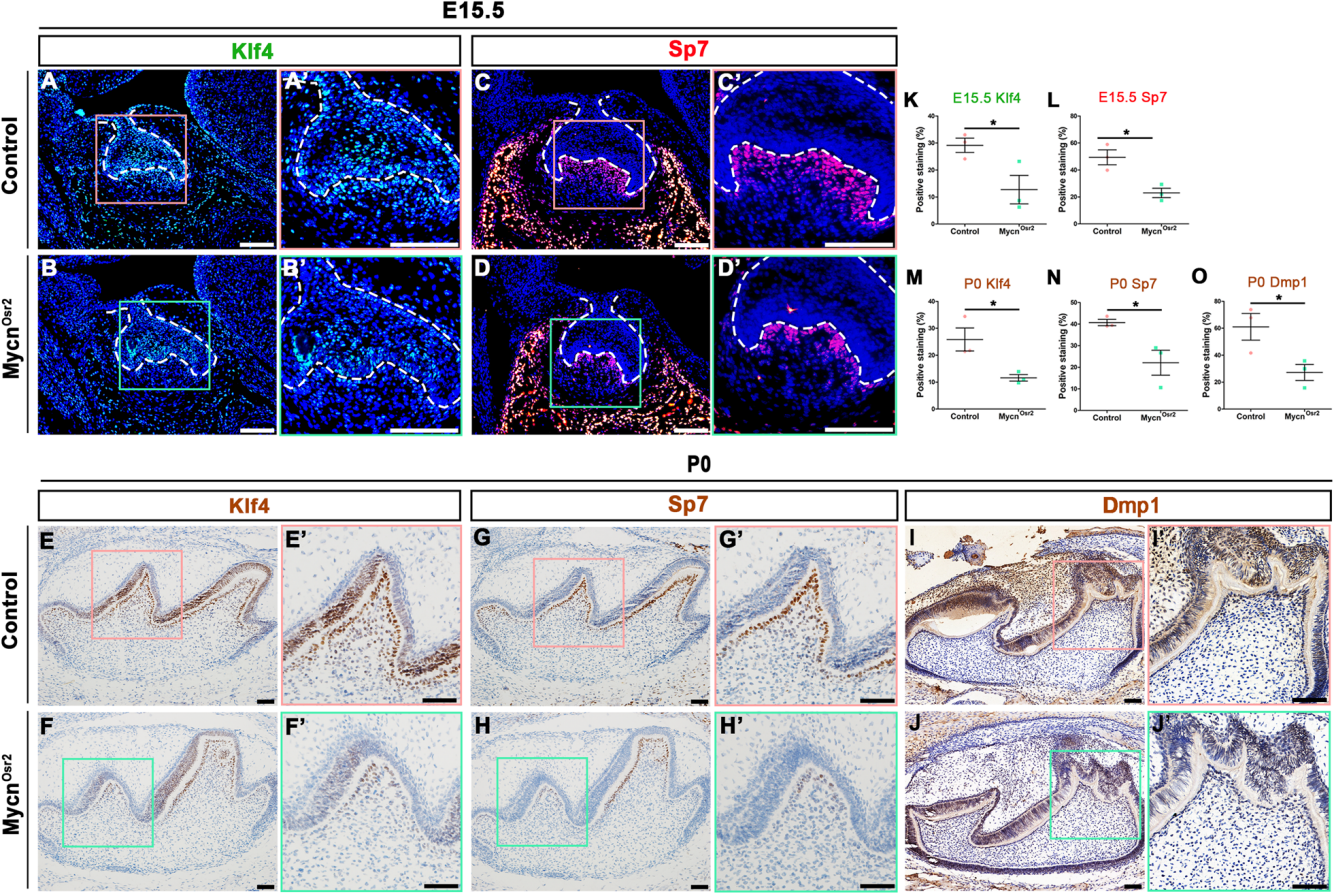
Mycn loss downregulated the expression of Klf4, Sp7 and Dmp1 in vivo. (**A–D’**) Representative immunofluorescence staining shows downregulation of Klf4 (**A–B’**) and Sp7 (**C–D’**) in mutants compared with the controls at E15.5. Dotted lines indicate the epithelial compartment of the tooth germ. (**E–J’**) Representative immunohistochemistry staining shows downregulation of Klf4 (**E–F’**), Sp7 (**G–H’**) and Dmp1 (**I–J’**) in mutants compared with the controls at P0. Scale bars: 50 μm. (**K–O**) Quantifications of the immunofluorescence and immunohistochemistry staining show significantly downregulated expression of Klf4, Sp7 and Dmp1 in mutants compared with the controls. *p < 0.05

**Fig. 6:**
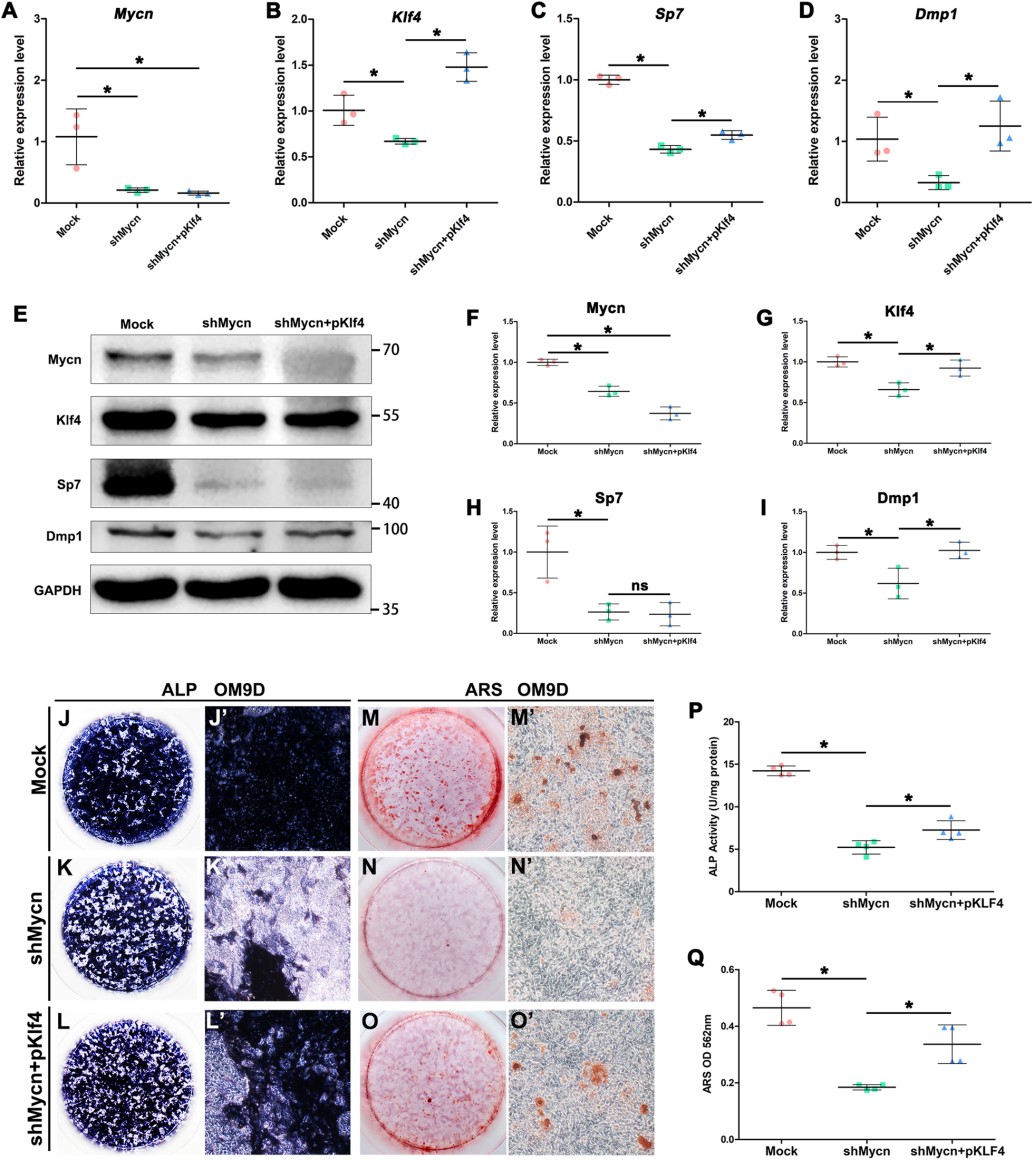
Overexpression of *Klf4* partially recovered the odontoblastic differentiation ability of Mycn-deficient mDPCs. (**A–D**) Quantitative PCR results show that while *Mycn* deficiency led to downregulated *Klf4*, *Sp7* and *Dmp1* mRNA expression, forced expression of *Klf4* in *shMycn* mDPCs upregulated *Sp7* and *Dmp1* mRNA expression levels compared with the *shMycn* group. (**E**) Representative western blots show upregulated expression of Dmp1 in *shMycn* mDPCs overexpressing Klf4 compared with the *shMycn* group. (**F–I**) Quantification of the western blots. The images used for western blotting quantitative analysis are included in Additional file 1: Fig. S3. (**J–O’**) Representative images of alkaline phosphatase (ALP) and Alizarin red staining (ARS) staining show that overexpression of *Klf4* partially rescued the odontoblastic differentiation and mineralization capacity of *shMycn* mDPCs. (**P**) Quantification of ALP activity shows significantly upregulated ALP activity in *shMycn* mDPCs overexpressing *Klf4* compared with *shMycn* mDPCs. (**Q**) Quantification of ARS shows significantly upregulated ARS activity in *shMycn* mDPCs overexpressing *Klf4* compared with *shMycn* mDPCs. *p < 0.05. *ns* not statistically significant

To uncover the regulatory mechanism of disrupted odontoblastic differentiation of dental papilla cells after loss of *Mycn*, CUT&Tag analysis was performed. CUT&Tag analysis is a novel and highly sensitive method to identify transcription factor occupancy sites [28, 29] by which the direct targets of Mycn during odontoblast commitment were identified. In our analysis, 2865 annotated peaks were significantly enriched (Additional file 2: Table S3). The largest proportion of Mycn-occupancy sites was located in the promoter region (55.07%; Fig. 4A), verifying that as a transcription factor, the main function of Mycn is to bind promoters and to regulate the expression of protein-coding genes. The five most enriched de novo motifs identified by HOMER software are listed in Fig. 4B, along with the top three best matches (*Sp1*, *Klf7* and *Klf4*) of the first enriched motif (motif #1) to known motifs. Among these annotated peaks, *Klf4* was of particular concern to us because substantial in vivo and in vitro studies have shown that this gene is strongly associated with odontoblastic differentiation and dentinogenesis [9, 10]. We suspected that *Klf4* may play a role in the Mycn downstream regulation network during odontoblast commitment. We found that the promoter region (mm10: chr4: 55532636–55532835) of *Klf4* (mm10: chr4: 555527143–55532466) was significantly enriched (Fig. 4C). To test whether *Klf4* could function as a direct target of Mycn, we first conducted a dual luciferase assay. As shown in Fig. 4D, Mycn overexpression significantly upregulated the luciferase activity of the *Klf4* promoter, suggesting that Mycn could not only bind to the promoter region of *Klf4* but also positively regulate its transcriptional activity. Furthermore, the expression level of Klf4 and its direct downstream targets Sp7 and Dmp1 were tested in vivo and in vitro. At E15.5, the expression of Klf4 (Fig. 5A, B’, K) and Sp7 (Fig. 5C, D’, L) was markedly decreased in the dental mesenchymal cells of *Mycn*^*Osr2*^ mice, and this downregulation was maintained in the neonatal stage (Fig. 5E–H’, M, N). In addition, the other Klf4 direct target, Dmp1, which is also an odontoblastic mature marker, also exhibited significantly decreased expression in the dental mesenchymal cells of *Mycn*^*Osr2*^ mice at P0 (Fig. 5I, J’, O). In addition, significantly downregulated expression of Klf4, Sp7 and Dmp1 was also observed in *Mycn*-deficient mDPCs in vitro (Fig. 6E; The images used for western blotting quantitative analysis are included in Additional file 1: Fig. S3.). Taken together, our results suggested that Mycn may impact the commitment of dental papilla cells to the odontoblast lineage through direct regulation of *Klf4.*

### Overexpression of *Klf4* partially rescued odontoblastic differentiation defects in *Mycn*-deficient mDPCs

To identify whether *Klf4* could rescue the odontoblastic differentiation defect caused by *Mycn* loss, we cotransfected mDPCs with *shMycn* and *pKlf4*. As shown in Fig. 6, *Mycn* knockdown (Fig. 6A, E, F) led to significantly downregulated mRNA and protein expression levels of *Klf4* (Fig. 6B, E, G), *Sp7* (Fig. 6C, E, H) and *Dmp1* (Fig. 6D, E, I) in vitro. However, upon forced expression of *Klf4* in *shMycn* cells, the mRNA expression levels of *Sp7* and *Dmp1* and the protein expression level of *Dmp1* were significantly upregulated compared with those in the *shMycn* group. The results of ALP (Fig. 6J–L’, P) and ARS (Fig. 6M–O’, Q) assays showed significantly increased mineralization capacity after forced gain of *Klf4* in *Mycn* knockdown mDPCs. As expected, the odontoblastic differentiation defect in *Mycn*-deficient mDPCs could partially be rescued by overexpressing *Klf4 *in vitro*.*

## Discussion

General elimination of *Mycn* led to embryonic lethality by E12.5 due to the maldevelopment of limb buds and internal organs. The abnormalities of the organs in the mutant embryos were closely related to the sites with high Mycn expression from E9.5 to E12.5. Myocardial cells, for example, express high levels of Mycn, and *Mycn* null mutants consistently form smaller hearts with thinner myocardia [30]. Thus, although there have been few previous studies in vitro and in vivo regarding the role of Mycn in tooth development, we hypothesized that Mycn may play an important role in the process based on the limited report demonstrating strong expression of *Mycn* in dental papilla cells during the embryonic period. Uniformly, as shown in our study, mesenchymal ablation of *Mycn* hindered odontoblastic differentiation and normal dentin formation. In addition, we found that Mycn was also expressed in dental epithelium. However, dental epithelial Mycn deletion under the control of the *K14* promoter seems to be no harm to tooth formation in mice, which may be partially explained by the fact that Mycn expression in dental epithelium is modest and its role in dental epithelium requires further analysis.

Mycn is a well-established proto-oncogene that has been proven to drive the development of numerous tumours, such as neuroblastoma [31], retinoblastoma [32], and rhabdomyosarcoma [33]. In addition to its impacts on tumorigenesis, Mycn affects the embryonic development of many organs, mostly by managing cell proliferation [34] and differentiation [35]. However, Mycn unexpectedly showed little ability to control the proliferation and apoptosis of dental mesenchymal cells in the present study. In contrast, elimination of *Mycn* in dental mesenchymal cells led to misshapen odontoblasts and to subsequent deficiency in odontoblastic mineralization and calcium deposition. Our results suggest that Mycn participates in dentin development by coordinating the commitment of dental papilla cells to the odontoblast lineage.

**Fig. 7:**
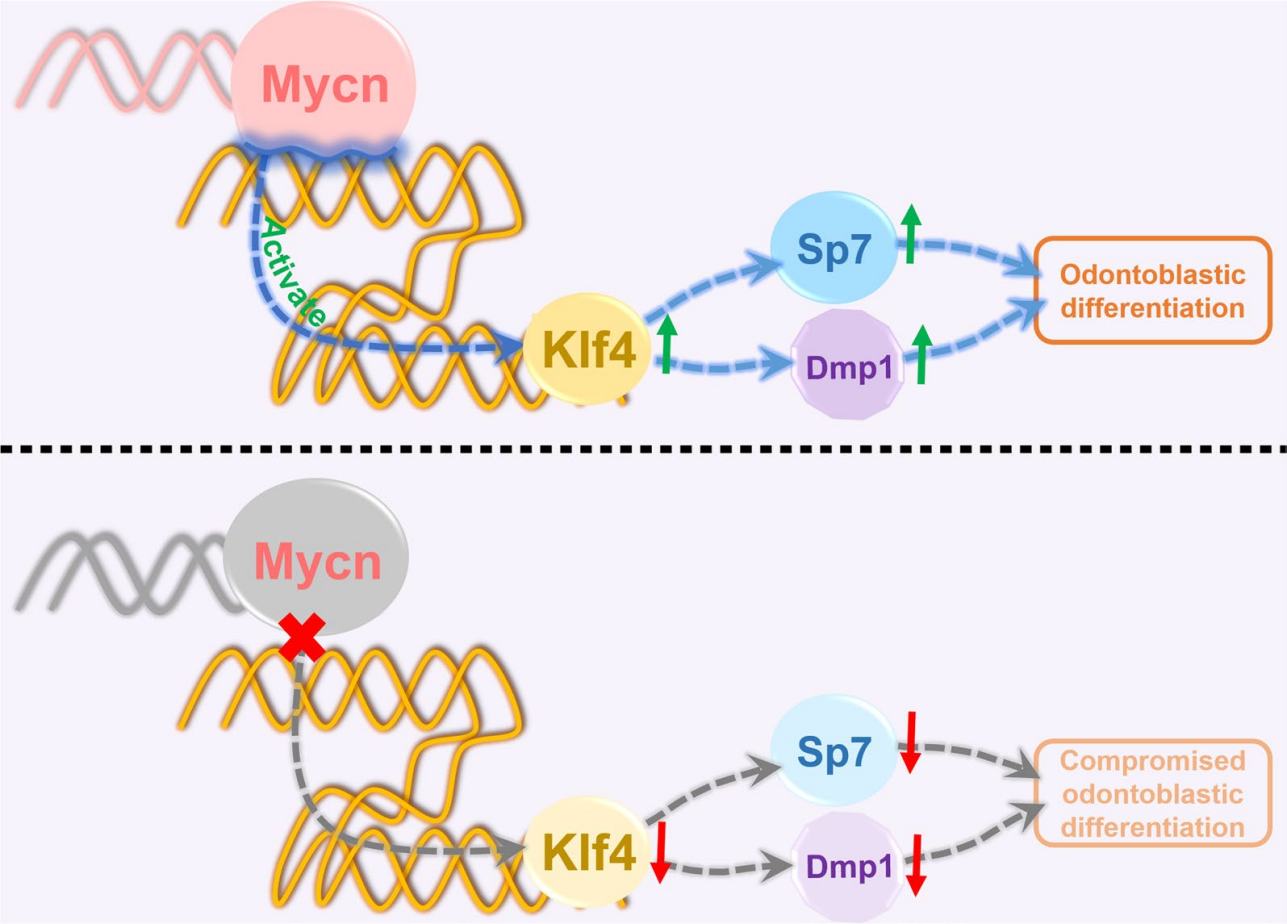
Schematic representation of the possible mechanism of *Mycn* in dentinogenesis. Mycn promotes odontoblast commitment of dental papilla cells by directly regulating *Klf4*

To reveal the possible mechanism by which Mycn governs odontoblastic differentiation, we performed CUT&Tag analysis, and *Klf4* was identified as one of the potential direct downstream targets of Mycn during dentinogenesis. Klf4 belongs to the three-zinc finger Krüppel-related family [36] and is well known for its multiple functions in physiological and pathological processes [37]. Klf4 has been reported to be involved in the development of diverse organs, including the intestine, eye, skin, bone, teeth and so on [38]. Similar to Mycn, Klf4 was expressed in both epithelial and mesenchymal cells in tooth germ. A previous study [10] showed that Klf4 could bind to the promoter regions of *Dmp1* and *Sp7* to control odontoblast differentiation and dentin formation. Specific ablation of *Klf4* in cranial neural crest cells resulted in decreased dentin density and thickness, which is highly similar to the phenotype of our *Mycn*^*Osr2*^ mutant mice. In our study, *Mycn* deficiency downregulated the expression of Klf4, Sp7 and Dmp1 in vivo and in vitro. It is plausible that, based upon our integrated methods, Mycn promotes odontoblast commitment of dental papilla cells and dentin formation by directly regulating *Klf4* (Fig. 7). However, overexpression of *Klf4* only partially recovered the odontoblastic differentiation ability of *Mycn*-deficient mDPCs in vitro in our study. Further studies are required to identify other critical factors involved in the Mycn-regulation network during dentinogenesis, which would provide novel insights into dentin hypoplasia and bioengineered dentin regeneration.

## Conclusion

Mesenchymal Mycn modulates the odontoblastic commitment of dental papilla cells by directly regulating *Klf4*. Our study illustrated the role of Mycn in dentin development and enhances our general comprehension of the transcription factor networks involved in the dentinogenesis process. Thus, these results may provide new insight into dentin hypoplasia and bioengineered dentin regeneration.

## Supplementary Information

**Additional file 1**:** Table S1**. Primary and second antibodies used in the study.** Table S2**. PCR primers used in the study.** Figure S1**. (A–C’) Gross appearances of mandibular molars and incisors of control (A–C) and *Mycn*^*K14*^ mutant (A’–C’) mice at PN6W; scale bars: 200 μm. (D) Quantification of tooth volume from Micro-CT scans of mandibular first molar of controls and *Mycn*^*K14*^ mutant mice; n = 4; ns = not statistically significant.** Figure S2**. Proliferation and apoptosis showed no significant difference between the* Mycn*^Osr2^ mutant mice and the controls. (A–B’) Immunofluorescence staining showing BrdU (red) (A, A’) and Ki67 (green) (B, B’) positive cells in the mandibular first molar tooth germs of E15.5 mice. (C–C’) Immunofluorescence staining showing cleaved Caspase 3 (green) positive cells in the mandibular first molar tooth germs of E15.5 mice. (A’’, B’’ and C’’) Graphs showing percentages of BrdU (A’’), Ki67 (B’’) and cleaved Caspase 3 (C’’) positive cells within the mesenchyme of tooth germs in controls and mutants; n > 3; ns = not statistically significant.** Figure S3**. Images used for western blotting quantitative analysis.

**Additional file 2**. The results of enriched peaks identified by CUT&Tag analysis.

## Data Availability

The datasets supporting the results of this article are included within the article and its additional files.

## Abbreviations

*Klf4*: *Krüppel-like Factor 4*
mDPCs: Mouse dental papilla cells
*Mycn*^*Osr2*^: *Mycn*^*fl/fl*^; *Osr2*^*IresCre*^
*Mycn*^*K14*^: *Mycn*^*fl/fl*^; *K14*^*Cre*^
ALP: Alkaline phosphatase
ARS: Alizarin red staining
BMP: Bone morphogenetic protein
SHH: Sonic hedgehog
FGF: Fibroblast growth factor
Osx/Sp7: Osterix
Gata4: GATA-binding protein 4
Runx2: Runt-related transcription Factor 2
PRS: Pierre Robin sequence
OCT: Optical cutting temperature
PFA: Paraformaldehyde
EDTA: Ethylenediaminetetraacetic acid
H&E: Haematoxylin and eosin
PBS: Phosphate-buffered saline
DAB: Diaminobenzidine
X-ray: X-radiography
BrdU: 5-Bromo-2′-deoxyuridine
DMEM: Dulbecco’s modified Eagle’s medium
FBS: Foetal bovine serum
CM: Control medium
OM: Odontoblastic medium
MEM: Minimum essential medium
*shMycn*: ShRNA targeting *Mycn*
cDNA: Complementary DNA
RT–qPCR: Real‐time quantitative PCR
RIPA: Radioimmunoprecipitation assay
PVDF: Polyvinylidene difluoride
CUT&Tag: Cleavage under targets and tagmentation
