# Supplementary material for: Mesenchymal Mycn participates in odontoblastic lineage commitment by regulating Krüppel-like Factor 4 (Klf4) in mice
Source: Stem Cell Res Ther. 2022 Feb 22;13:78. doi: 10.1186/s13287-022-02749-8 (PMC8864903; doi:10.1186/s13287-022-02749-8)
Supplement: Supplementary file 1 — Additional file 1: Table S1. Primary and second antibodies used in the study. Table S2. PCR primers used in the study. Figure S1. (A–C’) Gross appearances of mandibular molars and incisors of control (A–C) and MycnK14 mutant (A’–C’) mice at PN6W; scale bars: 200 μm. (D) Quantification of tooth volume from Micro-CT scans of mandibular first molar of controls and MycnK14 mutant mice; n = 4; ns = not statistically significant. Figure S2. Proliferation and apoptosis showed no significant difference between the MycnOsr2 mutant mice and the controls. (A–B’) Immunofluorescence staining showing BrdU (red) (A, A’) and Ki67 (green) (B, B’) positive cells in the mandibular first molar tooth germs of E15.5 mice. (C–C’) Immunofluorescence staining showing cleaved Caspase 3 (green) positive cells in the mandibular first molar tooth germs of E15.5 mice. (A’’, B’’ and C’’) Graphs showing percentages of BrdU (A’’), Ki67 (B’’) and cleaved Caspase 3 (C’’) positive cells within the mesenchyme of tooth germs in controls and mutants; n > 3; ns = not statistically significant. Figure S3. Images used for western blotting quantitative analysis. [file 13287_2022_2749_MOESM1_ESM.docx]

**Mesenchymal Mycn Participates in Odontoblastic Lineage Commitment by Regulating** ***Krüppel-like Factor 4* (*Klf4*) in Mice**

Zhuo Huang^1^, Ruihuan Yang^1^, Ruyi Li^1^, Yining Zuo^1^, Fan Gu^1^, Miao He^1*^, Zhuan Bian^1*^

^1^ The State Key Laboratory Breeding Base of Basic Science of Stomatology (Hubei-MOST) and Key Laboratory of Oral Biomedicine Ministry of Education, School and Hospital of Stomatology, Wuhan University, Wuhan, Hubei, China.

^*^ These authors jointly supervised this work.

Correspondence and requests for materials should be addressed to Miao He (D.D.S., Ph.D. E-mail: [hemiao@whu.edu.cn](mailto:hemiao@whu.edu.cn)) or to Zhuan Bian (D.D.S., Ph.D. E-mail: [bianzhuan@whu.edu.cn](mailto:bianzhuan@whu.edu.cn)).

**Supplemental Table S1.** Primary and second antibodies used in the study.

| **Antibody** | **Stock** | **Company** | **Concentration** | **Application** |
| --- | --- | --- | --- | --- |
| Mycn | 84406S | Cell Signaling Technology | 1:100 | IF |
|  |  |  | 1:50 | CUT&Tag |
|  | ab16898 | Abcam | 1:500 | WB |
| BrdU | ab6326 | Abcam | 1:200 | IF |
| Ki67 | ab15580 | Abcam | 1:200 | IF |
| Cleaved Caspase 3 | 9664 | Cell Signaling Technology | 1:100 | IHC |
| Klf4 | A13673 | Abclonal | 1:100 | IF |
|  |  |  | 1:400 | IHC |
|  |  |  | 1:1500 | WB |
| Sp7 | ab209484 | Abcam | 1:400 | IF |
|  |  |  | 1:1000 | IHC |
|  |  |  | 1:1500 | WB |
| Dmp1 | 45525 | Novus Biologicals | 1:150 | IHC |
|  |  |  | 1:1000 | WB |
| GAPDH | M171-7 | MBL | 1:5000 | WB |
| Goat anti-Rabbit IgG H&L | Ab206-01 | Vazyme | 1:100 | CUT&Tag |
| Goat Anti-Mouse IgG H&L (HRP) | A0216 | Beyotime | 1:1000 | WB |
| Goat Anti-Rabbit IgG H&L (HRP) | A0208 | Beyotime | 1:1000 | WB |
|  |  |  | 1:50 | IHC |
| Anti-rabbit IgG (H+L) F(ab')2 Fragment (Alexa Fluor® 488 Conjugate) | 4412 | Cell Signaling Technology | 1:400 | IF |
| Anti-mouse IgG (H+L) F(ab')2 Fragment (Alexa Fluor® 555 Conjugate) | 4409 | Cell Signaling Technology | 1:400 | IF |

**Supplemental Table S2.** PCR primers used in the study.

| PCR Primer Name | Sequence (5’-3’) |
| --- | --- |
| p*Klf4*-F | GATTCTAGAGCTAGCGAATTCGCCACCATGAGGCAGCCACCTGGC |
| p*Klf4*-R | ATCCTTCGCGGCCGCGGATCCAAAGTGCCTCTTCATGTGTAAGG |
| pGL6-*Klf4*-F | AGCCTCGAGGAGCTCACGCGTGAAGTCCCTTTGAGTGGGTTGG |
| pGL6-*Klf4*-R | CAGTACCGGATTGCCAAGCTTCCGGGGAACTGCCGGCGG |
| *Mycn*-F | ATTGGGCTACGGAGATGCTG |
| *Mycn*-R | AGTCCTGAAGGATGACCGGA |
| *Klf4*-F | CATTAATGAGGCAGCCACCTG |
| *Klf4*-R | GAGAGAGTTCCTCACGCCAA |
| *Sp7*-F | GGAAGGGTGGGTAGTCATTTG |
| *Sp7*-R | TCCTCTCTGCTTGAGGAAGAAG |
| *Dmp1*-F | AAATGGCTCTCACAGGACGG |
| *Dmp1*-R | TGCTCTGACATCATCCCACG |
| *Actb*-F | TGAGCTGCGTTTTACACCCT |
| *Actb*-R | TTTGGGGGATGTTTGCTCCA |


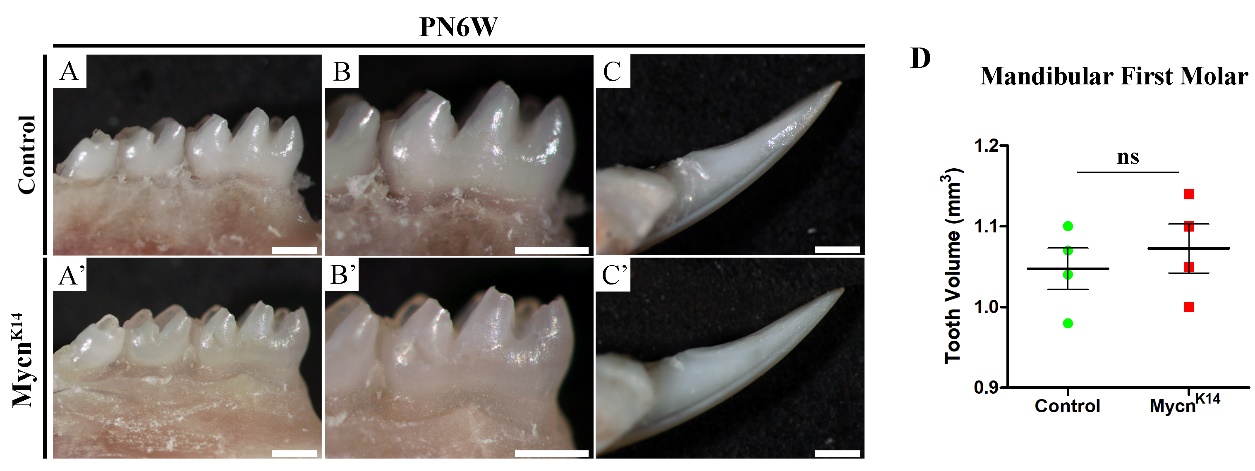


**Supplemental Figure S1.** (**A–C’**) Gross appearances of mandibular molars and incisors of control (**A-C**) and *Mycn^K14^* mutant (**A’-C’**) mice at PN6W; scale bars: 200 μm. (**D**) Quantification of tooth volume from Micro-CT scans of mandibular first molar of controls and *Mycn^K14^* mutant mice; n = 4; ns = not statistically significant.


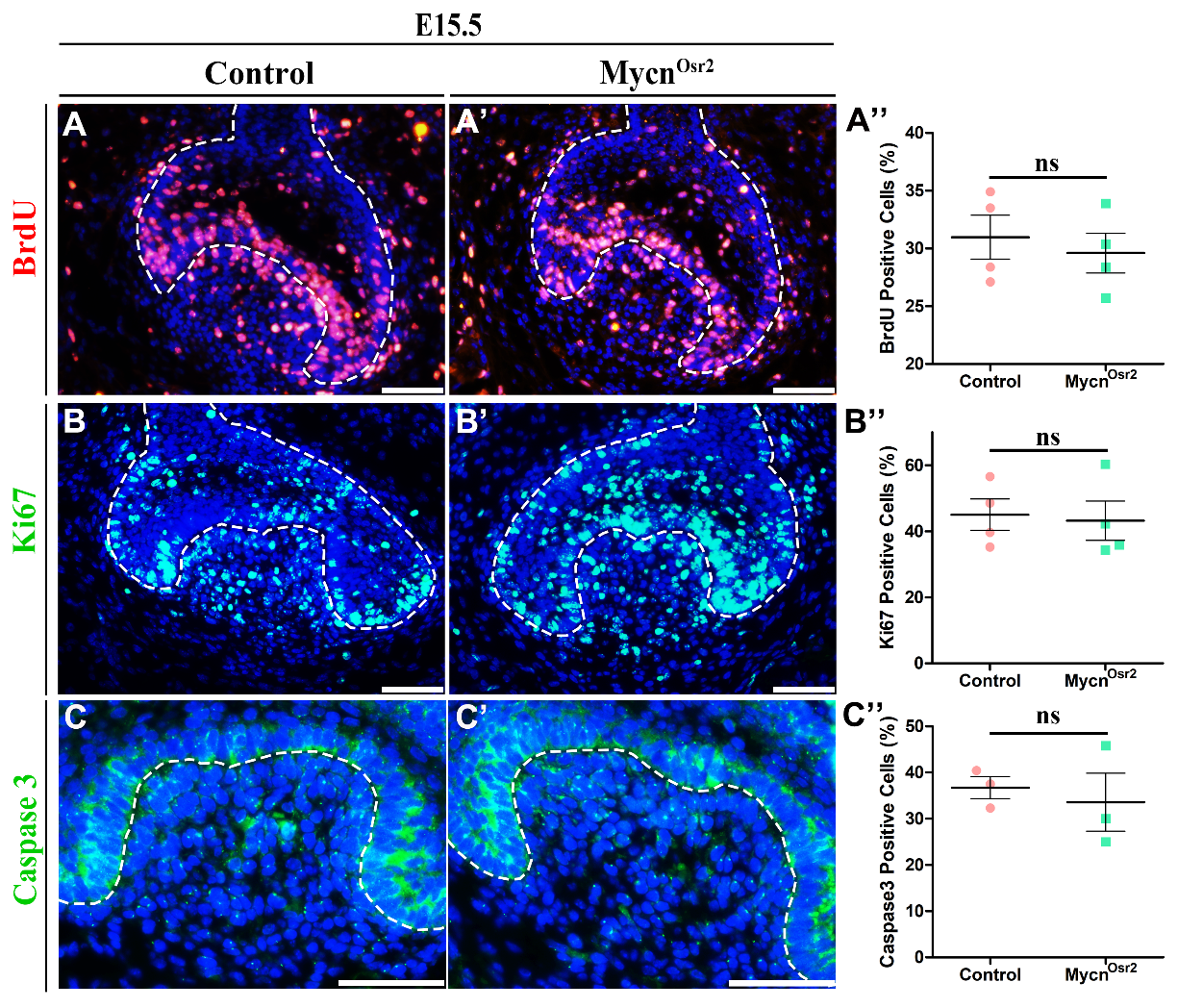


**Supplemental Figure S2.** Proliferation and apoptosis showed no significant difference between the *Mycn^Osr2^* mutant mice and the controls. (**A-B’**) Immunofluorescence staining showing BrdU (red) (**A, A’**) and Ki67 (green) (**B, B’**) positive cells in the mandibular first molar tooth germs of E15.5 mice. (**C-C’**) Immunofluorescence staining showing cleaved Caspase 3 (green) positive cells in the mandibular first molar tooth germs of E15.5 mice. (**A’’, B’’ and C’’**) Graphs showing percentages of BrdU (**A’’**), Ki67 (**B’’**) and cleaved Caspase 3 (**C’’**) positive cells within the mesenchyme of tooth germs in controls and mutants; n > 3; ns = not statistically significant.


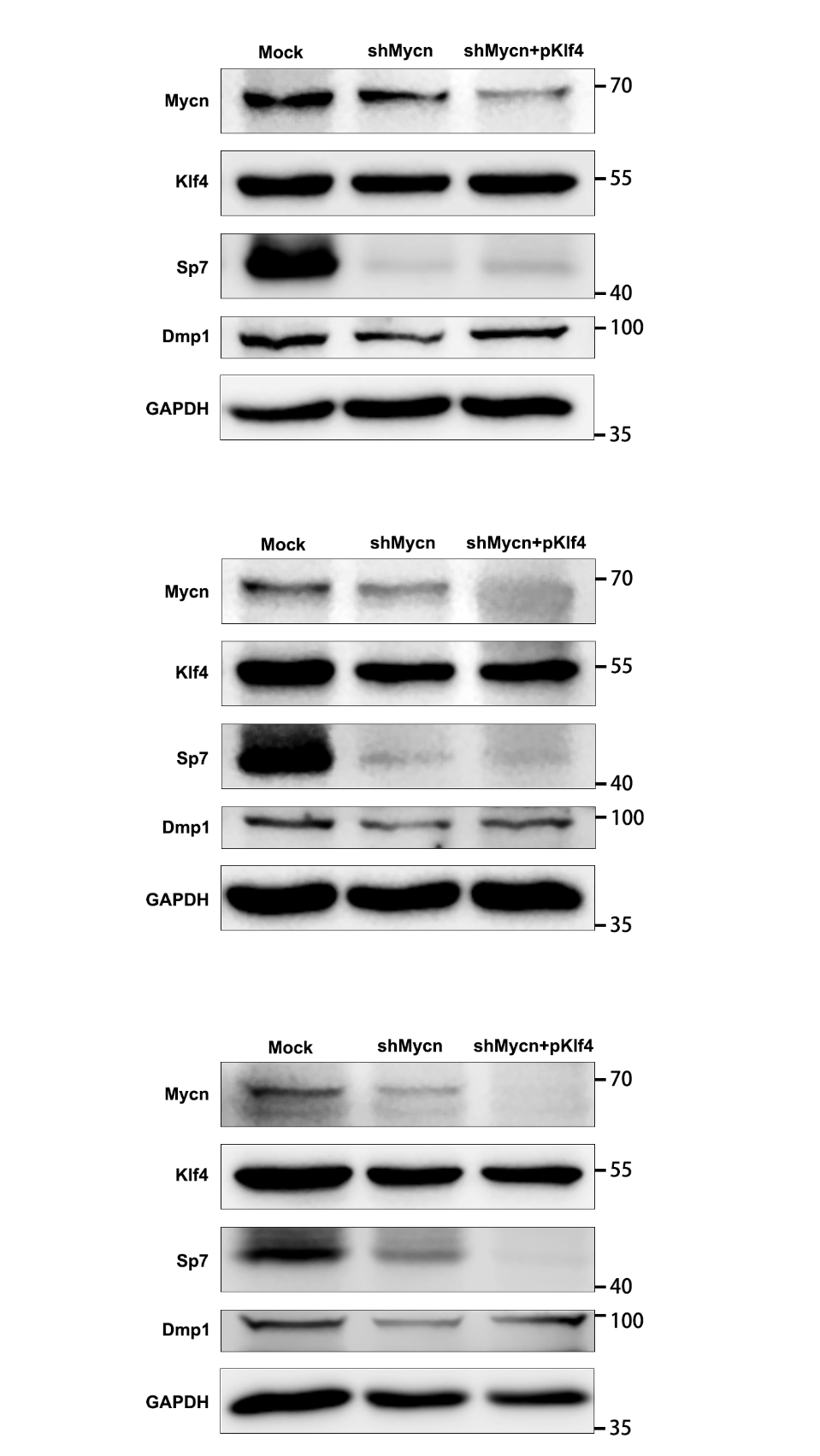


**Supplemental Figure S3.** Images used for western blotting quantitative analysis.
